# Engineering an asymmetric rhodamine dye suitable for developing ratiometric fluorescent probe

**DOI:** 10.1002/smo.20220002

**Published:** 2023-03-22

**Authors:** Feiyu Yang, Peng Lu, Tian‐Bing Ren, Xiao‐Bing Zhang, Lin Yuan

**Affiliations:** ^1^ State Key Laboratory of Chemo/Biosensing and Chemometrics College of Chemistry and Chemical Engineering Hunan University Changsha China

**Keywords:** fluorescent probe, imaging, nitroreductase, ratiometric probe, rhodamine

## Abstract

Fluorescent probes based on rhodamine skeleton are extensively used in biological imaging. However, the construction of ratiometric fluorescent probes based on the rhodamine skeleton without introducing additional fluorophores is still challenging. Herein, we propose an effective method to construct a rhodamine‐based ratiometric fluorescent probe through the regulation of electron cloud density. A ratiometric fluorescent probe RDQF‐RB‐NTR was successfully constructed for the detection of nitroreductase (NTR). RDQF‐RB‐NTR exhibits good sensitivity, high selectivity, and ratiometric response to NTR. Cell imaging experiments showed that RDQF‐RB‐NTR can rapidly and accurately detect the fluctuation of NTR in cells and difference of NTR levels between normal cells and cancer cells. In addition, RDQF‐RB‐NTR was successfully applied to the imaging of NTR in liver tissue slices, and we found that the level of NTR was upregulated in liver cirrhosis.

## Graphical abstract

We propose an effective strategy to construct rhodamine‐based ratiometric fluorescent probe through the regulation of electron cloud density in an asymmetric rhodamine dye. As proof of concept, a ratiometric fluorescent probe RDQF‐RB‐NTR was successfully constructed for the detection of NTR, which can be used for ratiometric imaging of NTR level in living cells and liver cirrhosis.

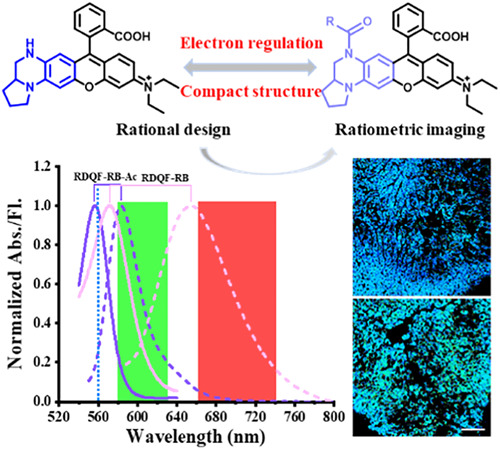

## 1 INTRODUCTION

**FIGURE 1:**
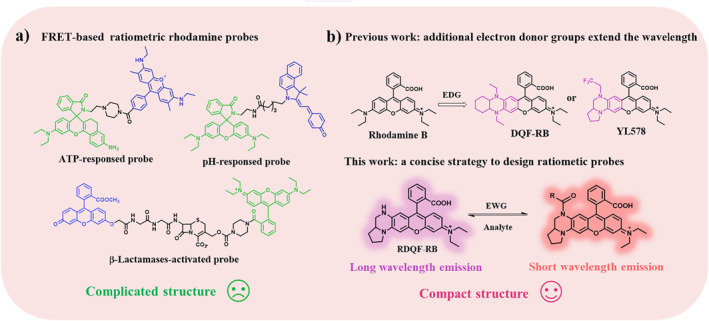
(a) Representative Förster (or fluorescence) resonance energy transfer (FRET)‐based ratiometric rhodamine fluorescent probes. (b) A strategy to develop a ratiometric fluorescent probe based on an asymmetric rhodamine dye.

Fluorescent probes have become effective tools for monitoring biomolecules in biological systems due to their high sensitivity, non‐invasive feature, real‐time imaging, and remarkable spatiotemporal resolution.[^1^, ^2^, ^3^, ^4^, ^5^, ^6^, ^7^] The performance of fluorescent probes are highly dependent on fluorescent dyes. So far, various fluorescent dyes have been reported for designing fluorescent probes.[^8^, ^9^, ^10^, ^11^, ^12^] Owing to its high fluorescent brightness and sharp absorption/fluorescence spectrum, rhodamine is one of the best effective fluorescent dyes for developing functionalized probes.[^13^, ^14^, ^15^, ^16^, ^17^] Currently, the traditional rhodamine‐based fluorescent probes can be divided into two categories: intensity‐based probes and ratiometric probes (Figure 1a). Intensity‐based rhodamine fluorescent probes mainly utilize the deprotection of amino group and the switch of spiro ring to restore the quinoid form, thus resulting in the enhancement of fluorescence.[^18^, ^19^, ^20^] However, fluorescent probes with single emission intensity are vulnerable to the external environment, such as probe concentration and excitation intensity. In contrast, ratiometric fluorescent probes based on dual channels can mitigate the external interference and provide more accurate results.[^21^, ^22^] Currently, Förster (or fluorescence) resonance energy transfer (FRET) is the most effective strategy to construct ratiometric fluorescent probes based on rhodamine.^[23]^ However, FRET‐based strategy remains constrained by the complex matching of energy donors, linkers, and energy acceptors. In addition, FRET‐based ratiometric fluorescent probes require complicated synthesis procedures.[^24^, ^25^, ^26^] The ratiometric probes based on a single fluorophore have simpler structures and higher synthesis efficiency compared with FRET‐based ratiometric probes. Recently, some ratiometric probes based on amino‐pyronine and benzo‐rosol have been reported.[^22^, ^27^, ^28^] In addition, a rosamine‐based probe for NTR was reported.^[28]^ However, the corresponding response spectrum and application were not given in this paper. Therefore, it is still a difficult task to design rhodamine‐based ratiometric fluorescent probes without introducing additional fluorophores.

Nitroreductase (NTR) is a reductase that catalyzes the reduction of aromatic nitro to amino.^[29]^ It plays an essential role in maintaining physiological redox balance and is associated with the development of various diseases and the progression of cancers.[^30^, ^31^, ^32^] Recently, some studies have indicated that NTR levels are generally upregulated in liver diseases, such as liver injury, fibrosis, and cancer.[^33^, ^34^, ^35^] However, the level of NTR in liver cirrhosis has rarely been studied. Although ultrasonography (US), computed tomography (CT), and magnetic resonance (MR) imaging are widely used for non‐invasive diagnosis of liver cirrhosis,[^36^, ^37^] biopsy remains the best method for the diagnosis of liver fibrosis. Therefore, the development of ratiometric probes capable of rapid and accurate detection of NTR levels in liver tissues would be essential to assess and diagnose cirrhosis.

Herein, we describe an effective strategy to construct rhodamine‐based ratiometric probes by changing the electron cloud density distribution in an asymmetric rhodamine (Figure 1b). In the presence of the electron‐withdrawing responsive group, the probe shows a small Stokes shift and emits short wavelength fluorescence. After reacting with the analyte, the dye with increased Stokes shift and red‐shifted emission wavelength is released. To verify the feasibility of this strategy, we then developed a ratiometric probe RDQF‐RB‐NTR for NTR detection. RDQF‐RB‐NTR exhibited good sensitivity, high selectivity, and ratiometric response to NTR. Cell imaging experiments showed that RDQF‐RB‐NTR can rapidly and accurately detect the fluctuation of endogenous NTR. In addition, RDQF‐RB‐NTR was applied to the ratiometric imaging of NTR in liver tissue slices, and we found that the level of NTR was upregulated in liver cirrhosis.

## 2 RESULTS AND DISCUSSION

### 2.1 Design and synthesis of the probe

In our previous works, we constructed an asymmetric dye DQF‐RB with long emission wavelength and improved Stokes shift by introducing a vibronic structure, 1,4‐diethyl‐decahydro‐quinoxaline.^[38]^ On this basis, in order to achieve the balance between fluorescence brightness and Stokes shift, we further developed a new type of asymmetric rhodamine with improved brightness by using fine‐tuned electron density of quinoxaline motif.^[39]^ Interestingly, the emission maximum shows a significant hypsochromic shift as the electron withdrawing (EW) ability of quinoxaline substituents increases, while the absorption maxima only shows a slight shift. That is, it is possible to develop a ratiometric fluorescent probe based on a single rhodamine dye, RDQF‐RB, by introducing a recognition unit with EW ability on quinoxaline (Figure 1b).

**FIGURE 2:**
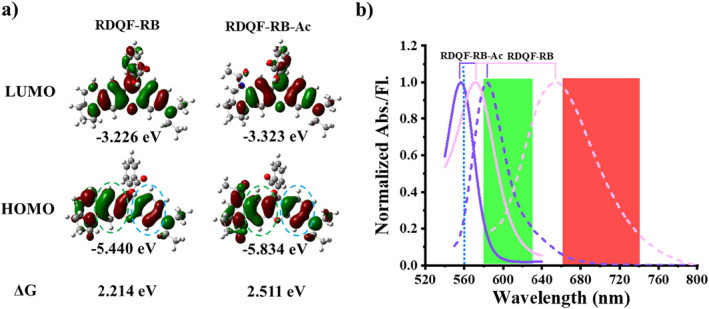
(a) Time‐dependent density functional theory (TD‐DFT) optimized molecular orbital plots (LUMO and HOMO) of RDQF‐RB and RDQF‐RB‐Ac. (b) Normalized absorption (solid line) and fluorescence (dash line) spectra of RDQF‐RB (pink) and RDQF‐RB‐Ac (purple) in PBS, *λ*_ex_: 560 and 530 nm, respectively. Dot line represents typical excitation wavelength (561 nm) in the confocal imaging system. The green and red rectangles represent two emission collecting channels in Olympus FV1000.

To verify whether the RDQF‐RB dye can be used to develop ratiometric probes, we first calculated the molecular frontier orbital distribution (HOMO & LUMO) and energy gap of RDQF‐RB and its acetylated derivative RDQF‐RB‐Ac (Figure 2a) through time‐dependent density functional theory. The π electrons on the LUMO of RDQF‐RB and RDQF‐RB‐Ac show relatively symmetrical electron distributions and similar orbital energies (−3.266 and −3.323 eV). However, compared with RDQF‐RB‐Ac, more electrons on the HOMO of RDQF‐RB are distributed on the quinoxaline moiety, and its HOMO orbital energy is also higher. Therefore, RDQF‐RB has a smaller energy gap (2.214 eV) than RDQF‐RB‐Ac (2.511 eV). We then synthesized rhodamine dyes, RDQF‐RB and RDQF‐RB‐Ac, and then tested their photophysical properties (Figure S1 and Table S1). Compared with RDQF‐RB‐Ac, the absorption maximum of RDQF‐RB shows a red‐shift of approximately 15 nm, while the Stokes shift increased by more than 50 nm, up to 84 nm. It is worth noting that the emission spectrum of these two dyes is well separated with emission shift about 65 nm (Figure 2b), indicating that the asymmetric rhodamine dye RDQF‐RB can be used to develop ratiometric fluorescent probes by introducing electron‐withdrawing responsive groups. As proof of concept, an NTR probe, RDQF‐RB‐NTR, was synthesized by introducing 4‐nitrobenzyl as the recognition and responsive moiety (Scheme S1) and the structure was confirmed using ESI and NMR (Supporting Information S1).

### 2.2 Probing nitroreductase in vitro

**FIGURE 3:**
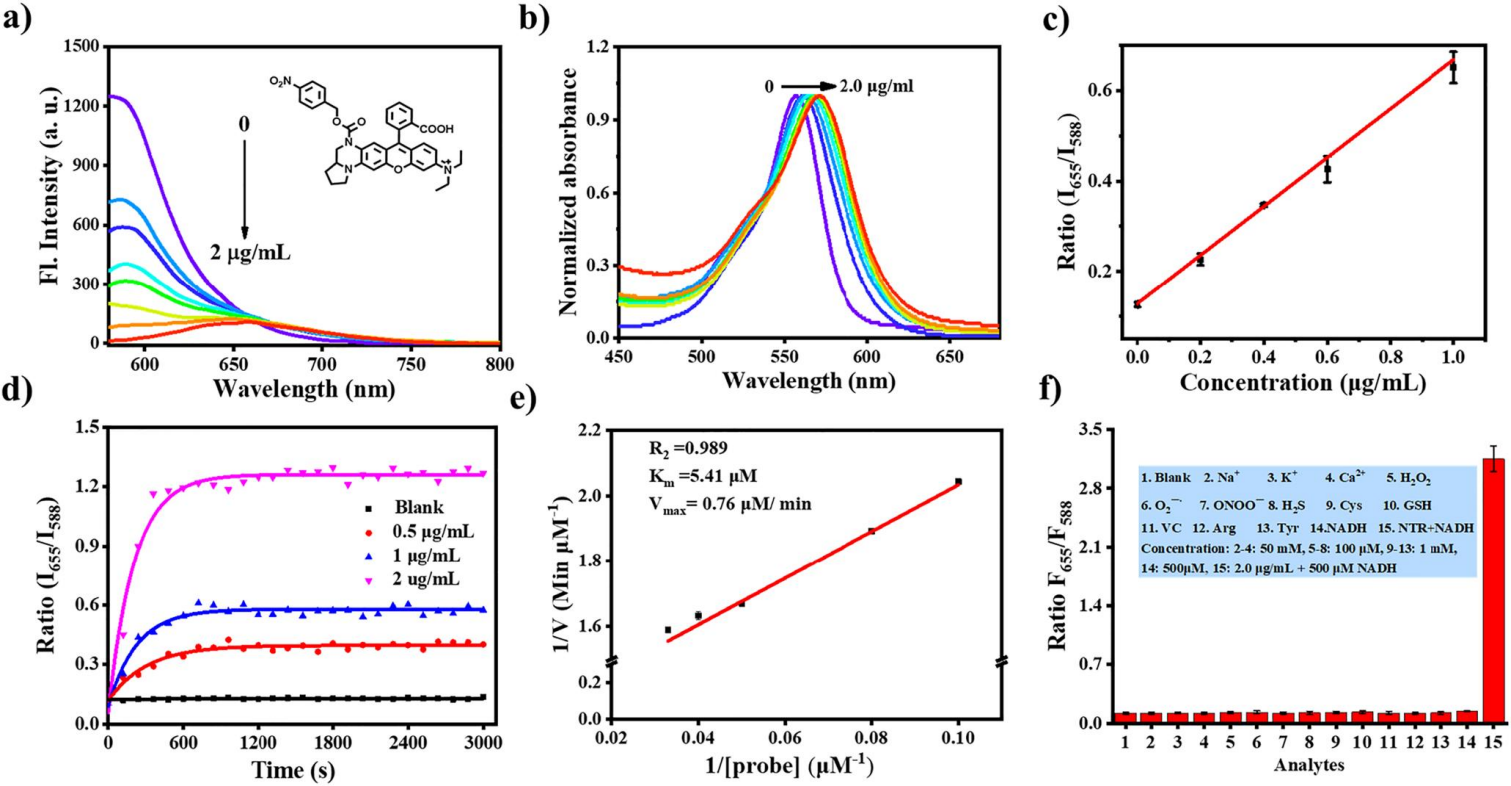
Fluorescence (a) and normalized absorbance (b) spectra of RDQF‐RB‐NTR responding to different concentrations of NTR, inset: structure of RDQF‐RB‐NTR. (c) Plot of *I*_655_/*I*_588_ with respect to [NTR] (0, 0.2, 0.4, 0.6, and 1.0 μg/mL). (d) Time‐dependent ratio (*I*_655_/*I*_588_) of RDQF‐RB‐NTR (5 μM) after reactions with different concentrations of NTR (0, 0.5, 1.0, and 2.0 μg/mL) in the presence of 500 μM NADH. (e) Line weaver‐Burk plot for the enzyme‐catalyzed reaction. The Michaelis‐Menten equation was described as: *V* = *V*_max_[probe]/(*K*_*m*_ + [probe]), where *V* is the reaction rate, [probe] is the probe concentration, and *K*_*m*_ is the Michaelis constant. Conditions: 2 μg/mL NTR, 10.0–30.0 μM of RDQF‐RB‐NTR. (f) Fluorescence intensity ratio (*I*_655_/*I*_588_) of RDQF‐RB‐NTR. 1: blank (probe: 5 μM); 2−4: 50 mM; 5−8: 100 μM; 9–13:1 mM; 14: 500 μM NADH; and 15: 2.0 μg/mL + 500 μM NADH. The medium was pH 7.4 PBS in all cases, and the data were taken after 20 min incubation at 37°C. *λ*_ex_ = 570 nm.

We then explored its response to NTR in vitro. As shown in Figure 3a, the probe RDQF‐RB‐NTR has strong fluorescence with maximum emission at 588 nm. With the increase in the concentration of NTR, the emission peak at 588 nm decreased obviously, and a new emission band appeared with a maximum peak at 655 nm. There was a good linearity between the emission ratio (*I*_655_/*I*_588_) and NTR concentration (Figure 3c) with the detection limit of 29 ng/mL. Meanwhile, the absorption peak gradually redshifted from the characteristic absorption wavelength of RDQF‐RB‐NTR to that of RDQF‐RB (Figure 3b), indicating that the p‐nitro benzyl moiety of the probe reacted with NTR and then was removed, which was verified by HRMS (Figure S2).

We then explored its sensitivity, affinity, and selectivity to NTR. We first measured the real‐time fluorescence changes of RDQF‐RB‐NTR after adding different concentrations of NTR. The results show that the probe RDQF‐RB‐NTR exhibited fast response to NTR and reached saturation within 10 min (Figure 3d). Accordingly, we calculated the Michaelis constant to be 5.41 μM (Figure 3e), indicating high affinity of RDQF‐RB‐NTR to NTR. Subsequently, we systematically studied the selectivity of the probe to metal ions (Na^+^, K^+^, and Ca^2+^), reactive oxygen species (H_2_O_2_, O_2_^.−^, and ONOO^−^), reactive sulfur species (H_2_S, Cys, and GSH), amino acids (arginine and tyrosine) and the reducing substance, vitamin C (VC). After incubation of RDQF‐RB‐NTR with other analytes for 20 min, the fluorescence intensity ratio of *I*_655_/*I*_588_ barely changed. However, the ratio was significantly enhanced under the coexistence of NTR (2 μg/mL) and NADH (500 μM), indicating good selectivity of RDQF‐RB‐NTR to NTR (Figure 3f). The above experiments demonstrate that RDQF‐RB‐NTR can realize the rapid and specific ratiometric detection of NTR. In addition, prior to application in vivo, CH_3_CN/PBS mixtures with different PBS fractions were utilized to assess the influence of polarity on the accuracy of results. RDQF‐RB‐NTR and RDQF‐RB showed a certain sensitivity to polarity, while the trend was consistent, which weakened the influence of polarity (Figure S3). Therefore, RDQF‐RB‐NTR is suitable for detecting NTR in a complex environment.

### 2.3 Probing nitroreductase in living cells

**FIGURE 4:**
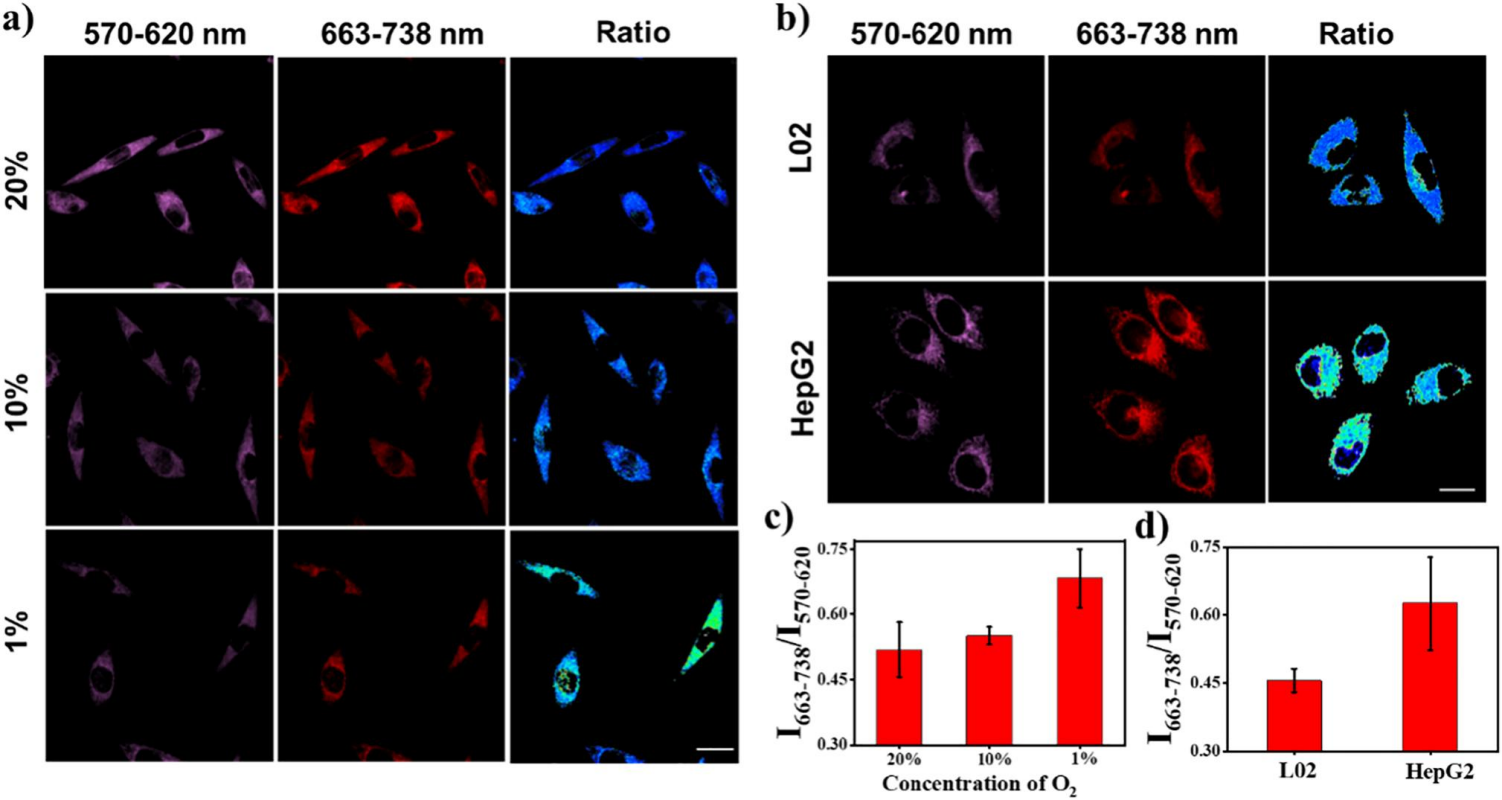
(a) Ratiometric detection of endogenous NTR in L02 cells under different concentrations of oxygen after treating with RDQF‐RB‐NTR (5 μM). (b) Image of probe RDQF‐NTR incubated with L02 cells and HepG2 cells. (c) Average fluorescence intensity ratio (*I*_663–738_/*I*_570–620_) of image (a). (d) Average fluorescence intensity ratio (*I*_663–738_/*I*_570–620_) of image (b). The probe was incubated for 20 min. *λ*_ex_ = 561 nm. Scale bar: 20 μm.

Inspired by the above favorable properties of RDQF‐RB‐NTR, we performed ratiometric imaging of NTR in biological systems. We first investigated the cytotoxicity of RDQF‐RB‐NTR through MTT (3‐(4,5)‐dimethylthiahiazo(‐z‐y1)‐3,5‐di‐phenytetrazoliumromide) assay (Figure S4). The results showed that the survival rate of L02 cells remained approximately 90% after incubation with 8 μM probe for 24 h, indicating appropriate biocompatibility of RDQF‐RB‐NTR. Colocalization experiments indicated that most probes were localized in mitochondria (Figure S5). Given numerous studies have shown that hypoxia would lead to an increase in NTR levels,[^40^, ^41^, ^42^] we then imaged intracellular NTR at different oxygen concentrations. The fluorescence intensity of the short‐wavelength channel from 570 to 620 nm (*I*_570–620_) was obviously reduced with the decrease in oxygen concentration, while the intensity of the long‐wavelength channel from 663 to 738 nm (*I*_663–738_) nearly unchanged (Figure 4a). Compared with normoxia conditions, the cellular fluorescence intensity ratio of I_663–738_/I_570–620_ increased obviously under hypoxic conditions, indicating that RDQF‐RB‐NTR was capable of detecting the upregulation of intracellular NTR induced by hypoxia. To verify the changes in fluorescence intensity were indeed induced by the response to NTR, we subsequently performed inhibitor experiments in L02 cells. After the treatment of dicumarol (DC), an inhibitor of NTR, the ratio of *I*_663–738_/*I*_570–620_ was significantly reduced, even lower than the control group (Figure S6), which might be attributed to the strong inhibitory effect of DC on endogenous NTR. The above results indicated that RDQ‐NTR can specifically detect endogenous NTR in cells.

It has been reported that cancer cells overexpress NTR due to active cellular activity and high levels of anaerobic respiration.[^43^, ^44^] We then tried to explore the difference of NTR levels between hepatoma cells (HepG2) and normal liver cells (L02) under normoxia conditions. Compared to L02 cells, HepG2 cells exhibited higher ratio of *I*_663–738_/*I*_570–620_ (Figure 4b), indicating higher level of NTR in cancer cells. These experiments verified that RDQF‐RB‐NTR can detect not only the fluctuation of NTR in the same cells but also the difference of NTR levels in different kinds of cells.

### 2.4 Probing nitroreductase in cirrhosis tissues

**FIGURE 5:**
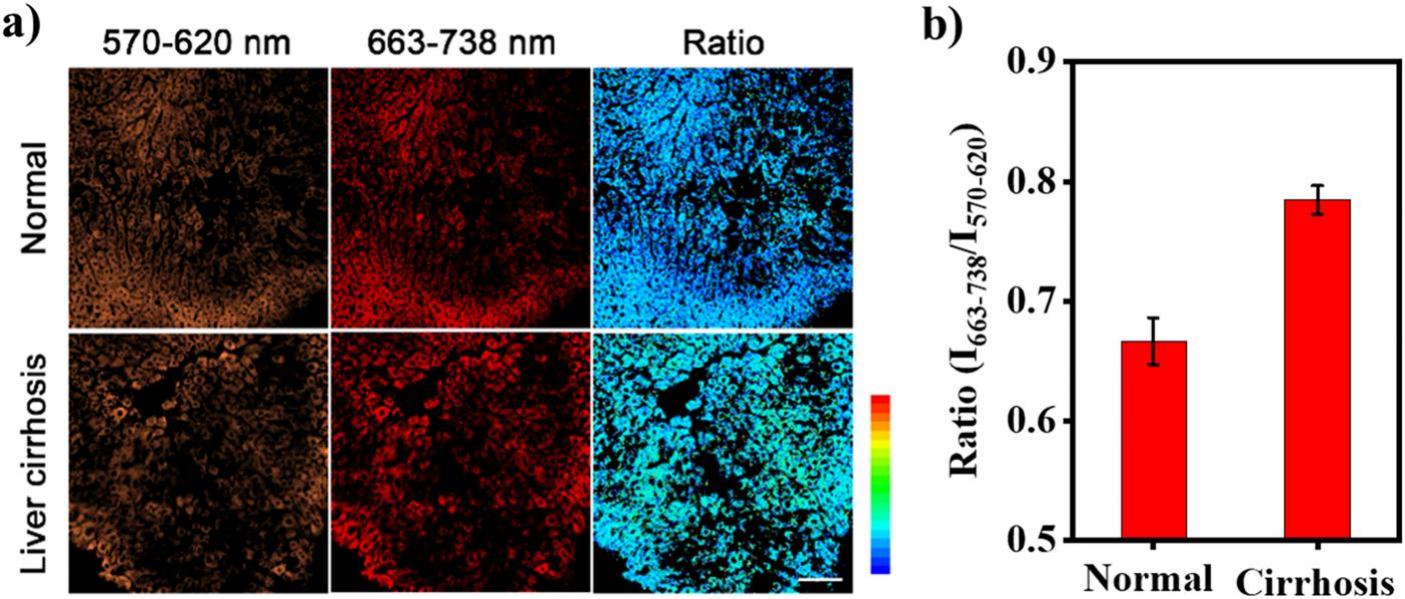
(a) Ratiometric detection of exogenous nitroreductase (NTR) in normal and cirrhosis liver tissues after treating with RDQF‐RB‐NTR (5 μM) for 20 min. (b) Average fluorescence intensity ratio (*I*_663–738_/*I*_570–620_) of image (a). *λ*_ex_ = 561 nm. Scale bar: 100 μm.

In liver diseases, there is a certain degree of hypoxia, resulting in the upregulation of NTR.^[45]^ Therefore, the level of NTR might be of a reference value in the diagnosis of liver cirrhosis. Considering that RDQF‐RB‐NTR can sensitively detect endogenous NTR in cells, we further applied RDQF‐RB‐NTR to detect NTR in liver cirrhosis. The mouse model of liver cirrhosis was induced by CCl_4_ according to the literature.^[46]^ After mice were sacrificed, liver tissue sections were incubated with RDQF‐RB‐NTR for 20 min followed by confocal imaging. Compared with the control group, the fluorescence intensity of the short‐wavelength channel in the cirrhosis group decreased significantly, while the long‐wavelength channel remained nearly unchanged (Figure 5a). Thus, the ratio of *I*_663–738_/*I*_570–620_ increased obviously. These results indicated that the level of NTR in liver cirrhosis tissue is higher than that in normal liver tissue, demonstrating the ability of RDQF‐RB‐NTR to detect NTR in liver tissues.

## 3 CONCLUSION

In summary, we have presented an effective method for developing the ratiometric probe based on an asymmetric rhodamine dye. Using this approach, we synthesized a NTR probe, RDQF‐RB‐NTR, and investigated its ability for ratiometric detection of NTR in vitro and in vivo. RDQF‐RB‐NTR exhibited low toxicity, good sensitivity, and high selectivity. The cell imaging experiments showed that RDQF‐RB‐NTR can rapidly and accurately detect the fluctuation of NTR in cells and distinguish the difference of NTR levels between normal cells and cancer cells. In addition, RDQF‐RB‐NTR can be used for ratiometric imaging of NTR in liver tissue slices, and we found that the level of NTR was upregulated in liver cirrhosis. The successful construction and application of RDQF‐RB‐NTR prove the effectiveness of the strategy. We believe that this strategy would greatly facilitate the construction of ratiometric probes and provide solutions for defects of existing probes in applications.

## CONFLICT OF INTEREST STATEMENT

The authors declare no conflicts of interest.

## ETHICS STATEMENT

All animal procedures were performed in accordance with protocol No. SYXK (Xiang) 2018‐0006 approved by the Hunan Provincial Science and Technology Department, and experiments were approved by the Animal Ethics Committee of College of Biology (Hunan University).

[Correction added on 17 April 2023, after first online publication: Ethics Statement has been corrected.]

## Supporting information

Supplementary Material S1

## Data Availability

The data that support the findings of this study are available in the supplementary material of this article.

## References

[1] W. Xu , Z. Zeng , J. H. Jiang , Y. T. Chang , L. Yuan , Angew. Chem. Int. Ed. 2016, 55, 13658. 10.1002/anie.201510721 27571316

[2] H. Singh , K. Tiwari , R. Tiwari , S. K. Pramanik , A. Das , Chem. Rev. 2019, 119, 11718. 31724399 10.1021/acs.chemrev.9b00379

[3] K. J. Bruemmer , S. W. M. Crossley , C. J. Chang , Angew. Chem. Int. Ed. 2020, 59, 13734. 10.1002/anie.201909690 PMC7665898 31605413

[4] H. H. Han , H. Tian , Y. Zang , A. C. Sedgwick , J. Li , J. L. Sessler , X. P. He , T. D. James , Chem. Soc. Rev. 2021, 50, 9391. 34232230 10.1039/d0cs01183e

[5] S. Wang , W. X. Ren , J. T. Hou , M. Won , J. An , X. Chen , J. Shu , J. S. Kim , Chem. Soc. Rev. 2021, 50, 8887. 34195735 10.1039/d1cs00083g

[6] Y. L. Xu , C. L. Li , X. Ma , W. Tuo , L. Tu , X. P. Li , Y. Sun , P. J. Stang , Y. Sun , Proc. Natl. Acad. Sci. U. S. A. 2022, 119, e2209904119. 35914164 10.1073/pnas.2209904119 PMC9371697

[7] W. Tuo , Y. L. Xu , Y. F. Fan , J. Li , M. Q. Qiu , X. X. Xiong , X. Y. Lie , Y. Sun , Coord. Chem. Rev. 2021, 443, 214017.

[8] Z. Qin , T. B. Ren , H. Zhou , X. Zhang , L. He , Z. Li , X. B. Zhang , L. Yuan , Angew. Chem. Int. Ed. 2022, 61, e202201541. 10.1002/anie.202201541 35218130

[9] D. Cheng , J. J. Peng , Y. Lv , D. D. Su , D. J. Liu , M. Chen , L. Yuan , X. B. Zhang , J. Am. Chem. Soc. 2019, 141, 6352. 30897899 10.1021/jacs.9b01374

[10] S. Y. Kim , A. Podder , H. Lee , Y. J. Cho , E. H. Han , S. Khatun , J. L. Sessler , K. S. Hong , S. Bhuniya , Chem. Sci. 2020, 11, 9875. 34094247 10.1039/d0sc03795h PMC8162098

[11] T. B. Ren , Z. Y. Wang , Z. Xiang , P. Lu , H. H. Lai , L. Yuan , X. B. Zhang , W. H. Tan , Angew. Chem. Int. Ed. 2021, 60, 800. 10.1002/anie.202009986 32918358

[12] J. H. Liu , W. Zhang , C. M. Zhou , M. M. Li , X. Wang , W. Zhang , Z. Z. Liu , L. L. Wu , T. D. James , P. Li , B. Tang , J. Am. Chem. Soc. 2022, 144, 13586. 35793548 10.1021/jacs.2c03832 PMC9354259

[13] J. B. Grimm , T. Klein , B. G. Kopek , G. Shtengel , H. F. Hess , M. Sauer , L. D. Lavis , Angew. Chem. Int. Ed. 2016, 55, 1723. 10.1002/anie.201509649 PMC4736676 26661345

[14] H. Y. Li , X. H. Li , X. F. Wu , W. Shi , H. M. Ma , Anal. Chem. 2017, 89, 5519. 28436652 10.1021/acs.analchem.7b00503

[15] N. Lardon , L. Wang , A. Tschanz , P. Hoess , M. Tran , E. D'Este , J. Ries , K. Johnsson , J. Am. Chem. Soc. 2021, 143, 14592. 34460256 10.1021/jacs.1c05004

[16] Y. Zheng , Z. W. Ye , Z. J. Liu , W. Yang , X. F. Zhang , Y. J. Yang , Y. Xiao , Anal. Chem. 2021, 93, 7833. 34027666 10.1021/acs.analchem.1c00175

[17] K. Kawai , T. Hirayama , H. Imai , T. Murakami , M. Inden , I. Hozumi , H. Nagasawa , J. Am. Chem. Soc. 2022, 144, 3793. 35133144 10.1021/jacs.1c08485

[18] M. Beija , C. A. Afonso , J. M. Martinho , Chem. Soc. Rev. 2009, 38, 2410. 19623358 10.1039/b901612k

[19] L. Q. Chen , X. Wu , H. J. Yu , L. Wu , Q. Wang , J. J. Zhang , X. G. Liu , Z. Li , X. F. Yang , Anal. Chem. 2021, 93, 14343. 34643369 10.1021/acs.analchem.1c03877

[20] R. Obara , M. Kamiya , Y. Tanaka , A. Abe , R. Kojima , T. Kawaguchi , M. Sugawara , A. Takahashi , T. Noda , Y. Urano , Angew. Chem. Int. Ed. 2021, 60, 2125. 10.1002/anie.202013265 33096584

[21] X. Huang , J. Song , B. C. Yung , X. Huang , Y. Xiong , X. Chen , Chem. Soc. Rev. 2018, 47, 2873. 29568836 10.1039/C7CS00612H PMC5926823

[22] X. B. Zhou , Y. W. Liu , Q. Liu , L. Z. Yan , M. Xue , W. Yuan , M. Shi , W. Feng , C. J. Xu , F. Y. Li , Theranostics 2019, 9, 4597. 31367243 10.7150/thno.35322 PMC6643432

[23] L. Yuan , W. Y. Lin , K. B. Zheng , S. S. Zhu , Acc. Chem. Res. 2013, 46, 1462. 23419062 10.1021/ar300273v

[24] J. Zhang , Y. Shen , S. L. May , D. C. Nelson , S. Li , Angew. Chem. Int. Ed. 2012, 51, 1865. 10.1002/anie.201107810 22246698

[25] T. B. Ren , S. Y. Wen , L. Wang , P. Lu , B. Xiong , L. Yuan , X. B. Zhang , Anal. Chem. 2020, 92, 4681. 32098468 10.1021/acs.analchem.0c00506

[26] Q. Q. Bai , C. J. Yang , M. J. Yang , Z. Q. Pei , X. B. Zhou , J. X. Liu , H. W. Ji , G. Li , M. M. Wu , Y. L. Qin , Q. Wang , L. Wu , Anal. Chem. 2022, 94, 2901. 34989555 10.1021/acs.analchem.1c04806

[27] K. H. Kim , S. Singha , Y. W. Jun , Y. J. Reo , H. R. Kim , H. G. Ryu , S. Bhuniab , K. H. Ahn , Chem. Sci. 2019, 10, 9028. 31762981 10.1039/c9sc02287b PMC6855311

[28] M. C. Dai , Y. J. Reo , C. W. Song , Y. J. Yanga , K. H. Ahn , Chem. Sci. 2020, 11, 8901. 34123144 10.1039/d0sc03314f PMC8163444

[29] E. M. Williams , R. F. Little , A. M. Mowday , M. H. Rich , J. V. Chan‐Hyams , J. N. Copp , J. B. Smaill , A. V. Patterson , D. F. Ackerley , Biochem. J. 2015, 471, 131. 26431849 10.1042/BJ20150650

[30] S. Y. Park , S. A. Yoon , Y. Cha , M. H. Lee , Coord. Chem. Rev. 2021, 428, 213613.

[31] Y. Zhou , H. Y. Lv , H. X. Li , J. Y. Li , Y. F. Yan , F. Y. Liu , W. L. Hao , Z. M. Zhou , P. Wang , S. M. Zhou , Appl. Environ. Microbiol. 2021, 87, e0175821. 34613761 10.1128/AEM.01758-21 PMC8612283

[32] M. R. Li , Y. Zhang , X. J. Ren , W. C. Niu , Q. Yuan , K. Cao , J. C. Zhang , X. Y. Gao , D. D. Su , Chem. Commun. 2022, 58, 819. 10.1039/d1cc06577g 34928281

[33] Z. Zeng , J. Ouyang , L. Sun , C. Zeng , F. Zeng , S. Z. Wu , Anal. Chem. 2020, 92, 9257. 32530263 10.1021/acs.analchem.0c01596

[34] X. P. Fan , T. B. Ren , W. Yang , X. B. Zhang , L. Yuan , Chem. Commun. 2021, 57, 8644. 10.1039/d1cc02999a 34369955

[35] X. X. Zhang , F. Y. Yang , T. B. Ren , Y. X. Zheng , X. B. Zhang , L. Yuan , Chin. Chem. Lett. 2022, 107835. 10.1016/j.cclet.2022.107835

[36] M. F. Kircher , J. K. Willmann , Radiology 2012, 263, 633. 22623690 10.1148/radiol.12102394 PMC3359513

[37] J. F. Li , J. M. Wang , S. P. Yu , G. D. Yuan , S. Q. He , Hepatology 2020, 71, 392. 31222762 10.1002/hep.30819

[38] T. B. Ren , W. Xu , W. Zhang , X. X. Zhang , Z. Y. Wang , Z. Xiang , L. Yuan , X. B. Zhang , J. Am. Chem. Soc. 2018, 140, 7716. 29792690 10.1021/jacs.8b04404

[39] G. W. Jiang , T. B. Ren , E. D'Este , M. Y. Xiong , B. Xiong , K. Johnsson , X. B. Zhang , L. Wang , L. Yuan , Nat. Commun. 2022, 13, 2264. 35477933 10.1038/s41467-022-29547-3 PMC9046415

[40] J. R. Zheng , Y. Z. Shen , Z. Q. Xu , Z. W. Yuan , Y. Y. He , C. Wei , M. Er , J. Yin , H. Y. Chen , Biosens. Bioelectron. 2018, 119, 141. 30125874 10.1016/j.bios.2018.08.014

[41] S. Sarkar , H. Lee , H. G. Ryu , S. Singha , Y. M. Lee , Y. J. Reo , Y. W. Jun , K. H. Kim , W. J. Kim , K. H. Ahn , ACS Sens. 2020, 6, 148. 33334101 10.1021/acssensors.0c01989

[42] K. Hanaoka , Y. Kagami , W. Piao , T. Myochin , K. Numasawa , Y. Kuriki , T. Ikeno , T. Ueno , T. Komatsu , T. Terai , T. Nagano , Y. Urano , Chem. Commun. 2018, 54, 6939. 10.1039/c8cc02451k 29862387

[43] I. Martinez‐Reyes , N. S. Chandel , Nat. Rev. Cancer 2021, 21, 669. 34272515 10.1038/s41568-021-00378-6

[44] B. I. Reinfeld , M. Z. Madden , M. M. Wolf , A. Chytil , J. E. Bader , A. R. Patterson , A. Sugiura , A. S. Cohen , A. Ali , B. T. Do , A. Muir , C. A. Lewis , R. A. Hongo , K. L. Young , R. E. Brown , V. M. Todd , T. Huffstater , A. Abraham , R. T. O'Neil , M. H. Wilson , F. Xin , M. N. Tantawy , W. D. Merryman , R. W. Johnson , C. S. Williams , E. F. Mason , F. M. Mason , K. E. Beckermann , M. G. Vander Heiden , H. C. Manning , J. C. Rathmell , W. K. Rathmell , Nature 2021, 593, 282. 33828302 10.1038/s41586-021-03442-1 PMC8122068

[45] M. Fernandez , D. Semela , J. Bruix , I. Colle , M. Pinzani , J. Bosch , J. Hepatol. 2009, 50, 604. 19157625 10.1016/j.jhep.2008.12.011

[46] Y. C. Liu , L. L. Teng , C. Xu , H. W. Liu , S. Xu , H. W. Guo , L. Yuan , X. B. Zhang , Chem. Sci. 2019, 10, 10931. 32190249 10.1039/c9sc03628h PMC7066674

