# Supplementary material for: Engineering an asymmetric rhodamine dye suitable for developing ratiometric fluorescent probe
Source: Smart Mol. 2023 Mar 22;1(1):e20220002. doi: 10.1002/smo.20220002 (PMC12118171; doi:10.1002/smo.20220002)
Supplement: Supplementary file 1 — Supplementary Material S1 [file SMO2-1-e20220002-s001.docx]

Supporting Information

Engineering an Asymmetric Rhodamine Dye Suitable for Developing Ratiometric Fluorescent Probe

Feiyu Yang^1^, Peng Lu^1^, Tian-Bing Ren*, Xiao-Bing Zhang, Lin Yuan*

**Table of contents**

1. Materials and General Experimental Methods……………………………………… 2
2. Determination of Fluorescence Quantum Yield…………………………………….. 2
3. MTT Assays for Probe……………………………………………………………. 2-3
4. Cell Culture…………………………………………………………………………. 3
5. Establishment of Liver Cirrhosis Mice Model……………………………………… 3
6. Confocal Fluorescence Imaging…………………………………………………….. 3
7. Computational Methods…………………………………………………………….. 4
8. Synthesis of Compounds………………………………………………………….. 4-6
9. Supplemental Figures………………………………………………………….…. 6-9
10. NMR and ESI Data…………………………………………………………...…. 9-13
11. References…………………………….…………………………………………. 13

**1. Materials and General Experimental Methods**

Unless otherwise stated, all reagents were purchased from commercial suppliers and used without further purification. Solvents used were purified by standard methods prior to use. Twice-distilled water was used throughout all experiments. NTR, NADH and dicoumarin (DC) were purchased from Sigma. The NTR powder and NADH was dissolved into pure water to form aqueous solution and was divided into several parts for daily experiments. To keep the enzyme activity, all these solutions were stored at -80 °C before use according to a reported procedure.^[1]^ Dicoumarin was dissolved in DMSO. Mass spectra were performed using an LCQ advantage ion trap mass spectrometer from Thermo Finnigan. NMR spectra were recorded on a Bruker-400 spectrometer, using TMS as an internal standard. Photoluminescent spectra were recorded at room temperature with a HITACHI F7000 fluorescence spectrophotometer (1 cm standard quartz cell). TLC analysis was performed on silica gel plates and column chromatography was conducted over silica gel (mesh 100–200), both of which were obtained from the Qingdao Ocean Chemicals.

**2. Determination of fluorescence quantum yield.**

Fluorescence quantum yield for RDQF-RB and RDQF-RB-Ac was determined by using cresyl violet (Φ_F_ = 0.58 in ethanol)^[2]^ and rhodamine B (Φ_F_ = 0.65 in ethanol)^[3]^ as a fluorescence standard, respectively. The quantum yield was calculated using the following equation:

Φ_F(X)_= Φ_F(S)_(A_S_F_X_/A_X_F_S_) (n_X_/n_S_)^2^ (1)

Where Φ_F_ is the fluorescence quantum yield, A is absorbance, F is relative integrated fluorescence intensity, and n is the refractive index of the solvents used. Subscripts S and X refer to the standard and to the unknown, respectively.

**3. MTT Assays for Probe**

L02 cells (1 × 10^4^ cells/well) were placed in a flat-bottom 96-well plate in 100 μL of culture medium and incubated in 5% CO_2_ at 37 °C for 24 h. The cells were treated with different concentrations (0−8 μM) of RDQF-RB-NTR. After 24 h of incubation, MTT solution (5.0 mg/mL) was added into each well (0.5 mg/mL). Residual MTT solution was removed after 4 h, and then DMSO (100 μL) was added to each well to dissolve the formazan crystals. After the plates were shaken for 10 min, the absorbance values of the wells were recorded by use of a microplate reader at 490 nm. The cytotoxic effects (VR) of RDQF-RB-NTR were assessed by the following equation: VR = A/A_0_ × 100, where A and A_0_ represent absorbance of the experimental group and control group, respectively. The assays were performed in six sets for each concentration.

**4. Cell Culture**

L02 and HepG2 cells were cultured in high glucose Dulbecco's Modified Eagle Medium (DMEM, Hyclone) supplemented with 10% fetal bovine serum (FBS, BI), and 1% antibiotics (100 U/mL penicillin and 100 µg/mL streptomycin, Hyclone) at 37 °C and 5% CO_2_. Cells were carefully harvested and split when they reached 80% confluence to maintain exponential growth.

**5. Establishment of Liver Cirrhosis Mice Model**

Female KM (18-20 g) mice were divided into 2 groups on average; group 1: CCl_4_-0 day (control), group 2: CCl_4_-48 days. Mice were subcutaneously injected with 50 µL 40% concentration of CCl_4_ diluted with cotton seed oil for 48 days. In the meantime, the mice were maintained with 30% ethanol in water as drinking water. The mice were sacrificed after administration day, then the liver tissues were excised. Liver tissues were immediately incubated with RDQF-RB-NTR for 20 min at 37 °C.

**6. Confocal Fluorescence Imaging**

Confocal fluorescence imaging was carried out on an Olympus FV1000 laser scanning confocal microscope. In the whole confocal fluorescence imaging experiments, a semiconductor laser at 561 nm was served as the sole excitation light source for imaging of the cells and liver tissues incubated with RDQF-RB-NTR. The collection channels of 570-620 nm and 663-738 nm correspond to the emissions of RDQF-RB-NTR and RDQF-RB, respectively. RDQF-RB-NTR probe dissolved in DMSO was added to DMEM to yield 5 μM solution. The cells were incubated with the RDQF-RB-NTR for 20 min at 37 °C.

**7. Computational Methods**

To describe the singlet excited state of RDQF-RB and RDQF-RB-Ac, TD-DFT theoretical calculations were performed. All the calculations were carried out using the Gaussian 09 program package1. All the geometries of RDQF-RB and RDQF-RB-Ac were optimized at B3LYP/6-31+G(d) level using a CPCM solvation model with EtOH as the solvent.

8. Synthesis of compounds

Scheme S1 Synthetic route of RDQF-RB-NTR.

Synthesis of RDQF-RB. Compound 1 were synthesized based on the previous literature.^[4]^ 2-(4-(diethylamino)-2-hydroxybenzoyl)benzoic acid (344.0 mg, 1.1 mmol, 1.1 equiv.) and compound 1 (205.0 mg, 1.0 mmol, 1.0 equiv.) were dissolved in methanesulfonic acid (3.0 mL). The mixture was stirred and heated at 90 °C for 6 h. After the reaction completing monitored by TLC analysis, the mixture was poured into ice water, and then perchloric acid (0.5 mL) was added. The resulting precipitate was filtered off and washed with water (200 mL). After the sample dried, purification by silica gel chromatography using a CH_2_Cl_2_/EtOH mixture as the eluent gave compound RDQF-RB (310 mg, 68％). RDQF-RB: ^1^H NMR (400 MHz, CDCl_3_/CD_3_OD (8/2)) δ 8.18 (s, 1H, Ar H), 7.64 (s, 2H, Ar H), 7.19 (s, 2H, Ar H), 6.90 – 6.72 (m, 2H, Ar H), 6.59 (s, 1H, Ar H), 6.26 (d, J = 10.9 Hz, 1H, Ar H), 3.91 – 3.49 (m, 9H, CH_2_, CH), 2.28 (s, 2H, CH_2_), 2.17 (s, 1H, CH_2_), 1.68 – 1.54 (m, 1H, CH_2_), 1.34 – 1.25 (m, 6H, CH_3_). ^13^C NMR (101 MHz, CDCl_3_/CD_3_OD (8/2)) δ 156.56, 156.27, 154.63, 153.34, 146.27, 134.44, 134.41, 130.77, 130.59, 130.09, 129.70, 129.67, 129.31, 115.41, 113.36, 113.03, 105.27, 105.10, 95.45, 94.49, 58.31, 45.13, 43.81, 43.73, 29.55, 22.81, 11.77. HRMS: calcd for C_29_H_30_N_3_O_3_ 468.2282, found 468.2282.

Synthesis of RDQF-RB-Ac. RDQF-RB (50.0 mg, 88.3 μmol, 1.0 equiv.) and triethylamine (30.0 μL, 530.1 μmol, 6.0 equiv.) was dissolved into THF (10.0 mL). Acetyl chloride (25.0 μL, 353.4 μmol, 4.0 equiv.) was added dropwise and the mixture was stirred at r.t. for 1 h. Water (10.0 mL) was added carefully to the above mixture for quenching the reaction. The mixture was extracted with CH_2_Cl_2_ (20.0 mL) three times. The organic phases were washed with H_2_O containing 0.5 mL HClO_4_ for three times, dried, concentrated. And the crude was purified by column chromatography on silica gel chromatography using a CH_2_Cl_2_/EtOH mixture as the eluent and gave compound RDQF-RB-Ac (44 mg, 80%). RDQF-RB-Ac: ^1^H NMR (400 MHz, CDCl_3_/CD_3_OD (8/2)) δ 8.37 (d, J = 7.5 Hz, 1H, Ar H), 7.84 (t, J = 6.6 Hz, 2H, Ar H), 7.80 (s, 1H, Ar H), 7.45 (d, J = 6.8 Hz, 1H, Ar H), 7.29 (d, J = 9.4 Hz, 1H, Ar H), 7.06 (d, J = 7.3 Hz, 1H, Ar H), 7.01 (s, 1H, Ar H), 6.93 (s, 1H, Ar H), 3.89 (t, J = 10.3 Hz, 2H, CH_2_), 3.81 – 3.68 (m, 7H, CH, CH_2_), 2.45 – 2.37 (m, 2H, CH_2_), 2.24 (d, J = 7.9 Hz, 4H, CH_2_), 1.72 (d, J = 7.1 Hz, 1H, CH_2_), 1.42 (t, J = 5.5 Hz, 6H, CH_3_). ^13^C NMR (101 MHz, CDCl_3_/CD_3_OD (8/2)) δ 170.11, 169.34, 157.86, 157.45, 155.42, 155.22, 146.82, 146.57, 133.04, 131.32, 131.05, 130.08, 129.43, 113.98, 113.90, 113.82, 112.81, 95.88, 95.80, 58.75, 57.32, 45.73, 31.65, 29.90, 29.40, 23.06, 17.47, 13.69, 12.11. MS (ESI): calcd for C_31_H_32_N_3_O_4_ 510.24, found 510.46.

**Synthesis of RDQF-RB-NTR**. **RDQF-RB** (50.0 mg, 88.3 μmol, 1.0 equiv.) and p-nitrobenzyl chloroformate (57.0 mg, 0.26 mmol, 3.0 equiv.) were dissolved in 10 mL of anhydrous CH_2_Cl_2_. Then K_2_CO_3_ (55.0 mg, 0.35 mmol, 4.0 equiv.) was added and the solution was stirred for 1 h at room temperature under N_2_ environment. After concentration, the obtained crude product was purified by silica column chromatography using a CH_2_Cl_2_/EtOH mixture to afford purple solid product (38 mg, 56%). ^1^H NMR (400 MHz, CDCl_3_**/**CD_3_OD (8/2)) δ 8.31 (d, J = 8.4 Hz, 2H, Ar H), 8.22 (s, 1H, Ar H), 7.72 (s, 1H, Ar H), 7.55 (d, J = 6.8 Hz, 1H, Ar H), 7.50 (d, J = 4.9 Hz, 2H, Ar H), 7.24 (s, 2H, Ar H), 7.10 (d, J = 9.4 Hz, 1H, Ar H), 6.77 (d, J = 14.2 Hz, 2H, Ar H), 6.66 (d, J = 4.6 Hz, 1H, Ar H), 5.48 – 5.19 (m, 2H, CH_2_), 3.77 (s, 2H, CH_2_), 3.63 (d, J = 6.6 Hz, 5H, CH, CH_2_), 3.54 – 3.44 (m, 2H, CH_2_), 2.44 – 2.31 (m, 3H, CH_2_), 1.72 (s, 2H, CH_2_), 1.48 (m, 6H, CH_3_). HRMS: Calcd for C_37_H_35_N_4_O_7_ 647.2500, found 647.2500.

**9. Supplemental Figures**

|  | Solvent | | *λ_ab_* | | | | *λ_em_* | Stokes shift | | | *ε* | *Φ* |
| --- | --- | --- | --- | --- | --- | --- | --- | --- | --- | --- | --- | --- |
|  | |  | | (nm) | | (nm) | | | (nm) | (M^-1^cm^-1^) | |  |
| **RDQF-RB** | | DCM | | | 583 | 644 | | | 61 | 6.71×10^4^ | | 0.29 |
|  | | MeCN | | | 584 | 653 | | | 69 | 6.25×10^4^ | | 0.16 |
|  | | EtOH | | | 574 | 659 | | | 85 | 5.58×10^4^ | | 0.09 |
|  | | PBS | | | 571 | 655 | | | 84 | 6.65×10^4^ | | 0.06 |
| **RDQF-RB-Ac** | | DCM | | | 561 | 590 | | | 29 | 1.04×10^5^ | | 0.77 |
|  | | MeCN | | | 562 | 596 | | | 34 | 9.03×10^4^ | | 0.57 |
|  | | EtOH | | | 548 | 581 | | | 33 | 8.30×10^4^ | | 0.69 |
|  | | PBS | | | 556 | 590 | | | 34 | 8.88×10^4^ | | 0.44 |

**Table S1** Photophysical properties of RDQF-RB and RDQF-RB-Ac.


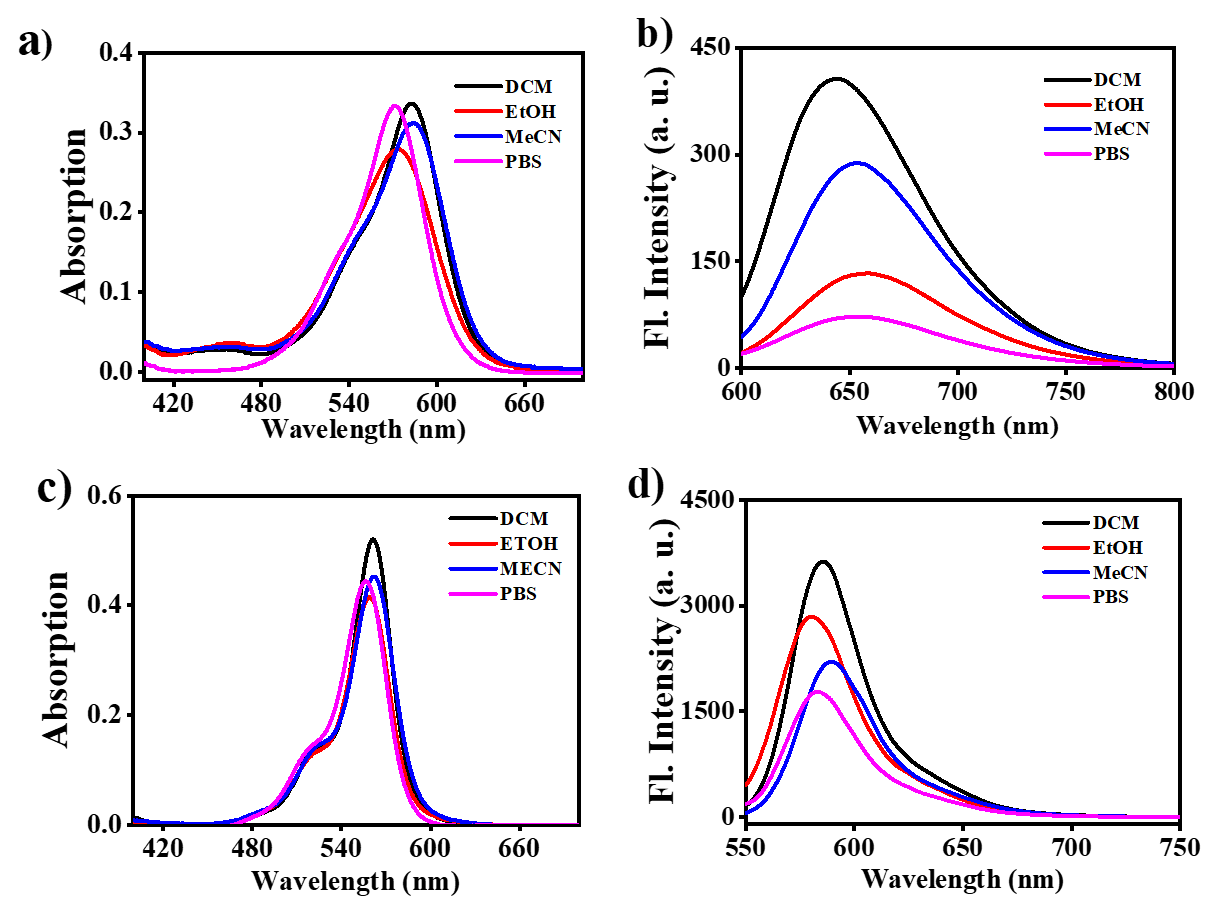


**Figure S1** Absorption and emission spectra of RDQF-RB (a, b), RDQF-RB-Ac (c, d) (5 μM) in various solvents (CH_2_Cl_2_, MeCN, EtOH, PBS buffer (10 mM)) at room temperature.


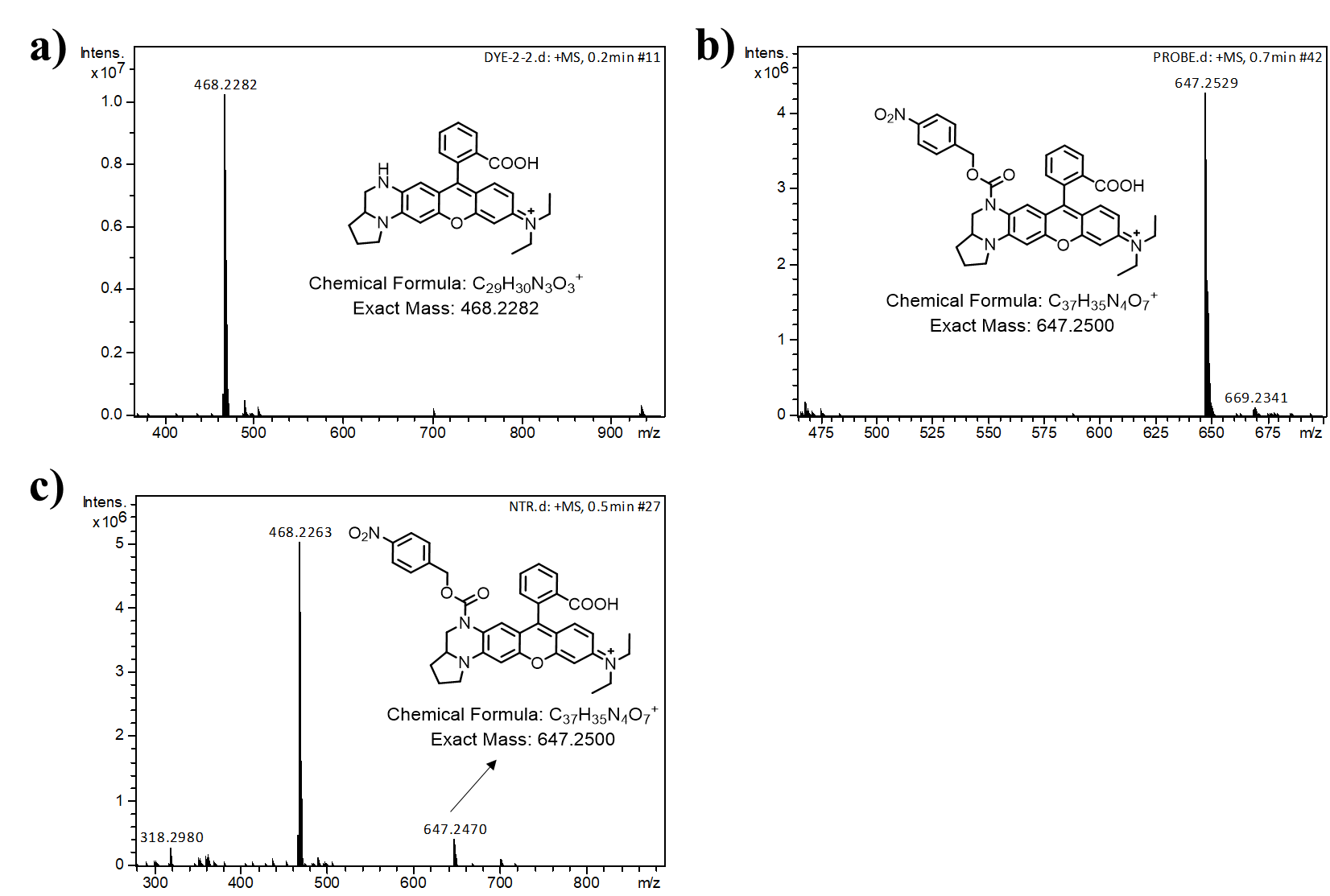


**Figure S2**. HR-MS spectrums of RDQF-RB (a), RDQF-RB-NTR (b), and RDQF-RB-NTR after response with 2.0 μg/ml NTR (c).


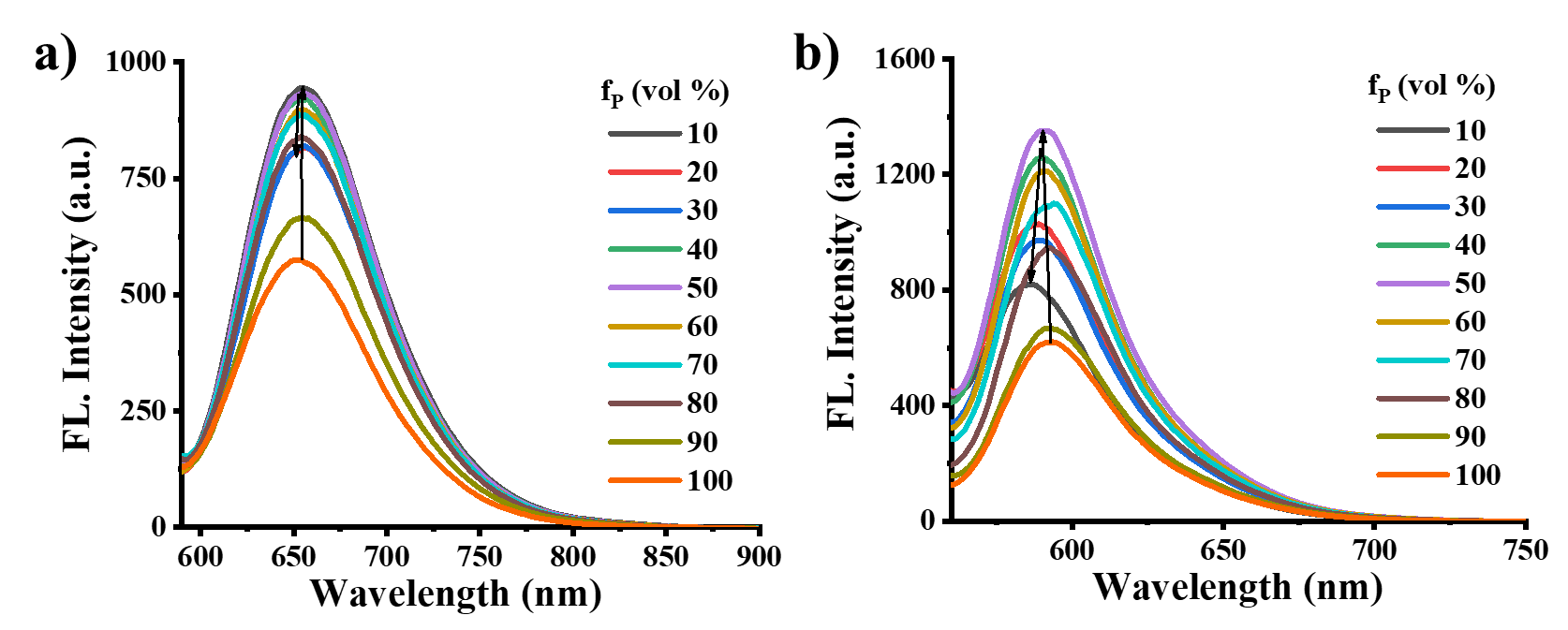


**Figure S3** Emission spectra of RDQF-RB-NTR (a) and RDQF-RB (b) (5 μM) in CH_3_CN/PBS mixtures with different PBS (pH 7.4, 10 mM, including 1% DMSO) fractions (f_P_); λ_ex_: 560 nm and 530 nm, respectively.


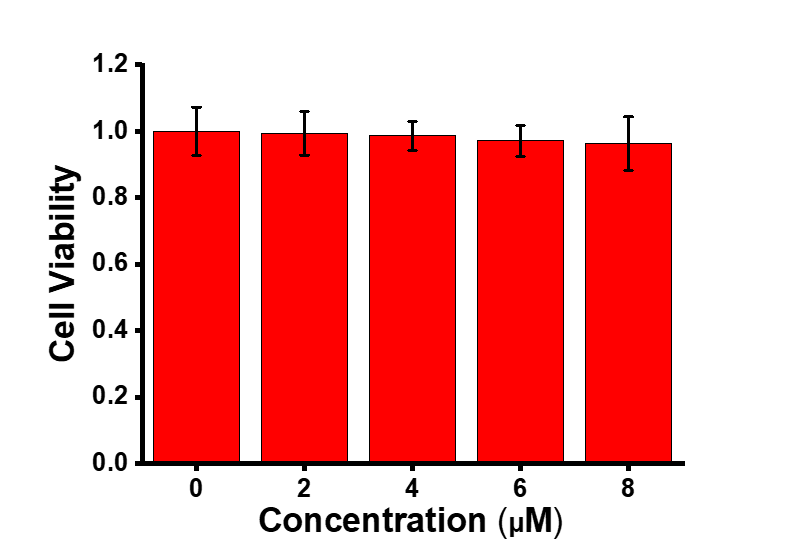


**Figure S4** Effects of RDQF-RB-NTR with varied concentrations (0-8 μM) on the viability of L02 cells. The viability of the cells without probe is defined as 100%. Data are expressed as mean ± SD of three parallel experiments.


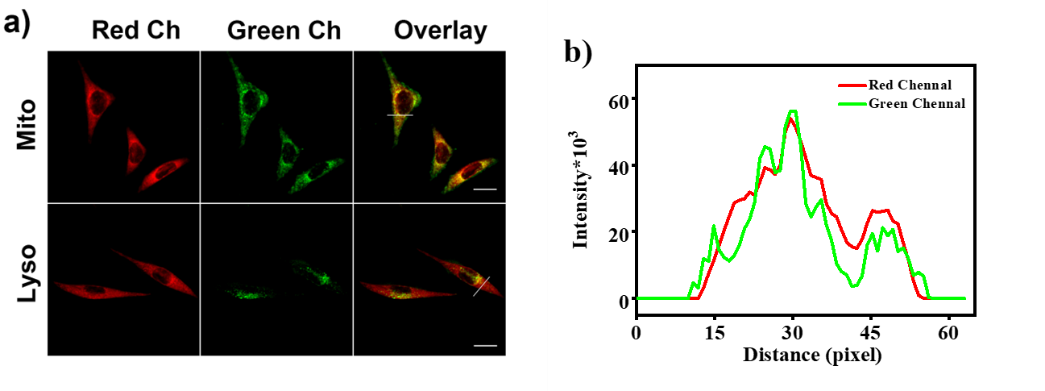


**Figure S5** (a) Confocal microscopy images of probe RDQF-RB-NTR with Mito-Tracker Green (0.5 μM) or Lyso-Tracker Green (0.5 μM) in live L02 cells. (b) Line contours of red and green channels in the mitochondrial localization group of images (a). Red channel of RDQF-NTR, at 663-738 nm, λ_ex_ = 561 nm. Green channel of Mito-Tracker Green and Lyso-Tracker Green at 500-550 nm, λ_ex_ = 488 nm.


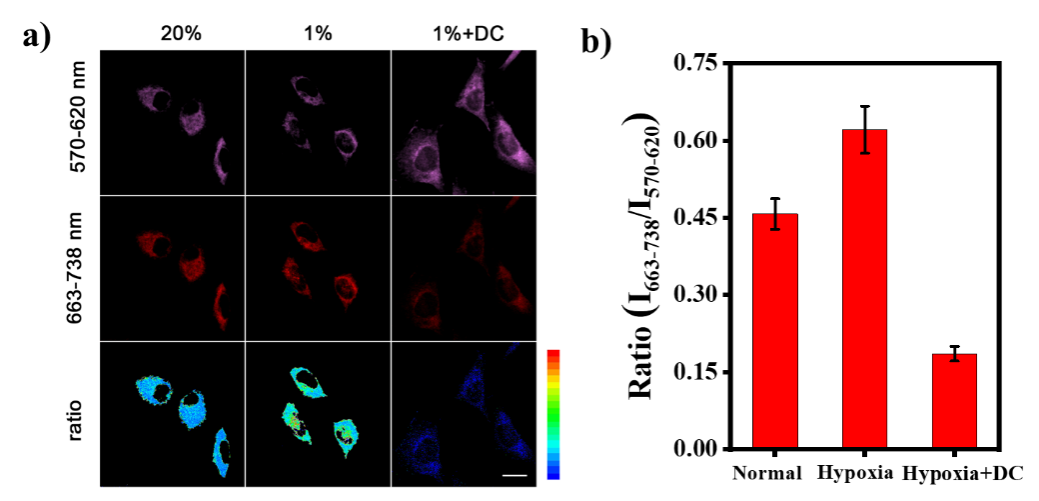


**Figure S6** (a) Confocal imaging of probe RDQF-RB-NTR in L02 cells under different oxygen concentrations (1% and 20% O_2_) or inhibitor conditions (1% O_2_ + DC). (b) Fluorescence intensity ratio of two channels under different conditions in image (a). The error line is the standard deviation (SD). λ_ex_ = 561 nm, scale bar: 20 μm.

**10. NMR and ESI Data**


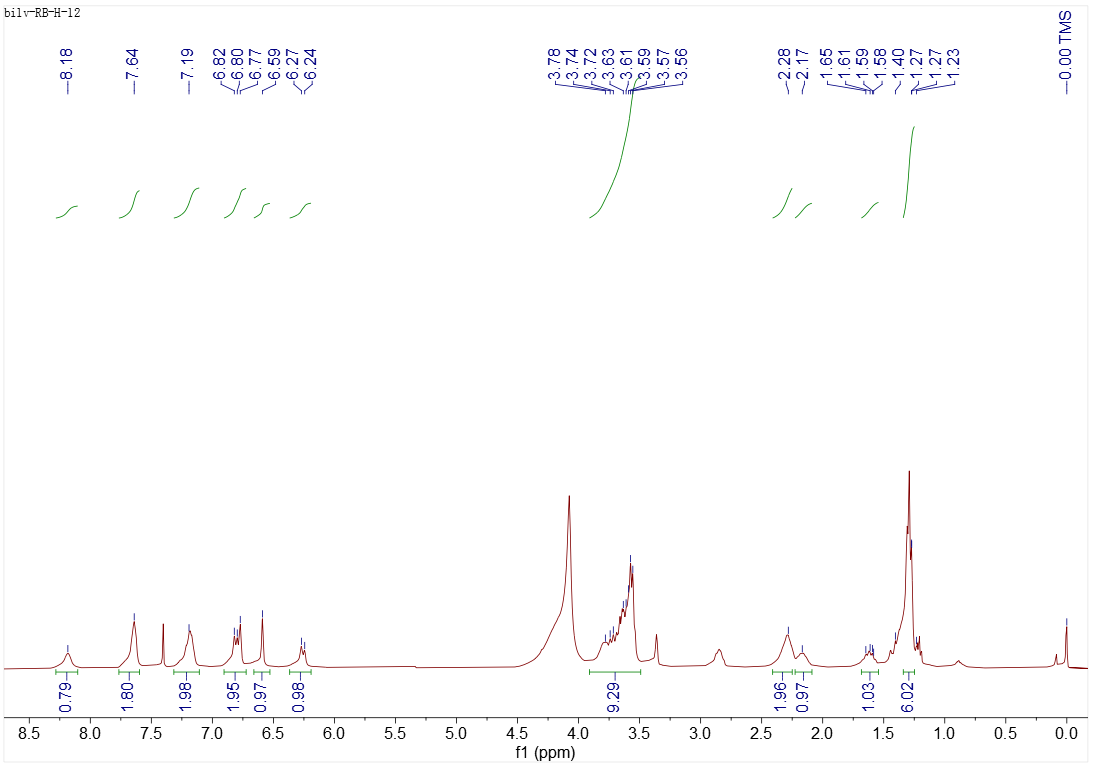


**Figure S7.** ^1^HNMR spectrum of RDQF-RB in CDCl_3_ containing 20% CD_3_OD.


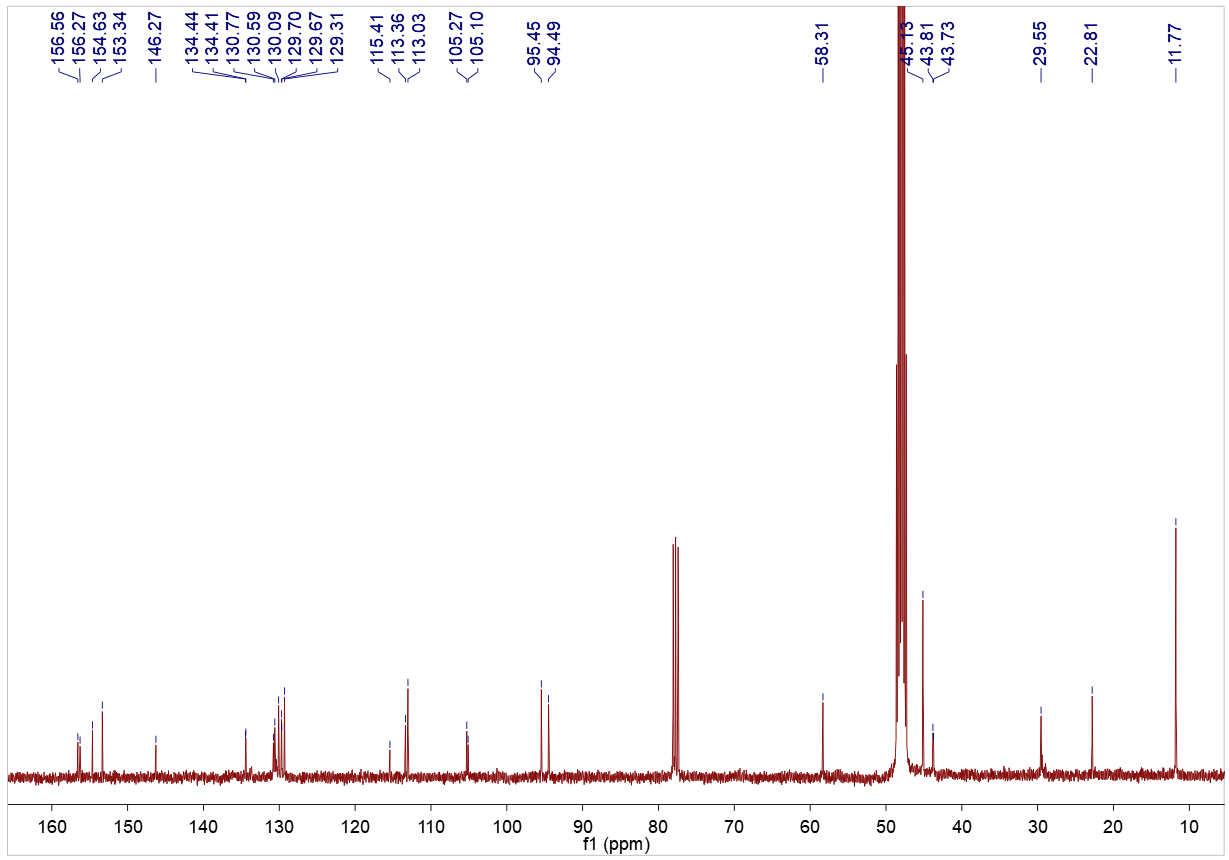


**Figure S8.** ^13^CNMR spectrum of RDQF-RB in CDCl_3_ containing 20% CD_3_OD.

**
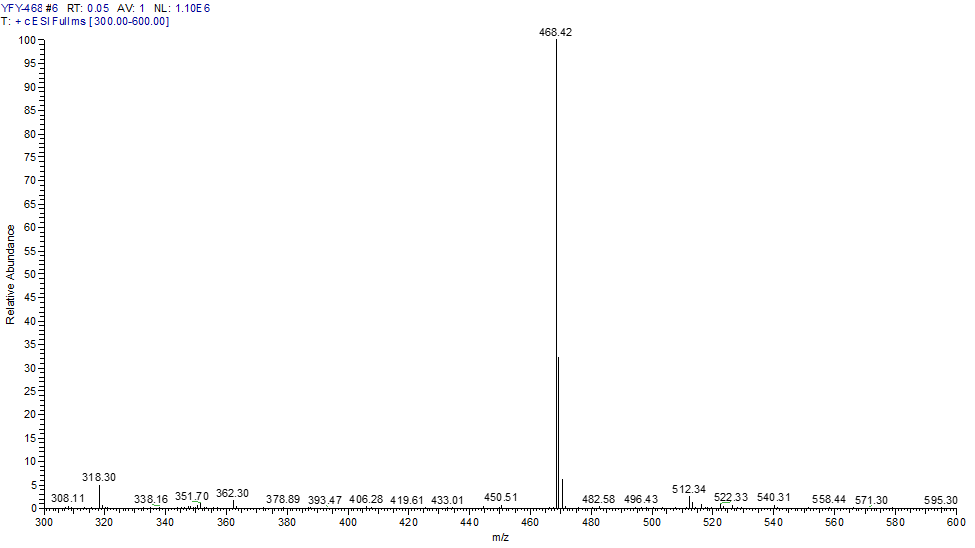
**

**Figure S9.** Mass spectrum (ESI) of RDQF-RB.


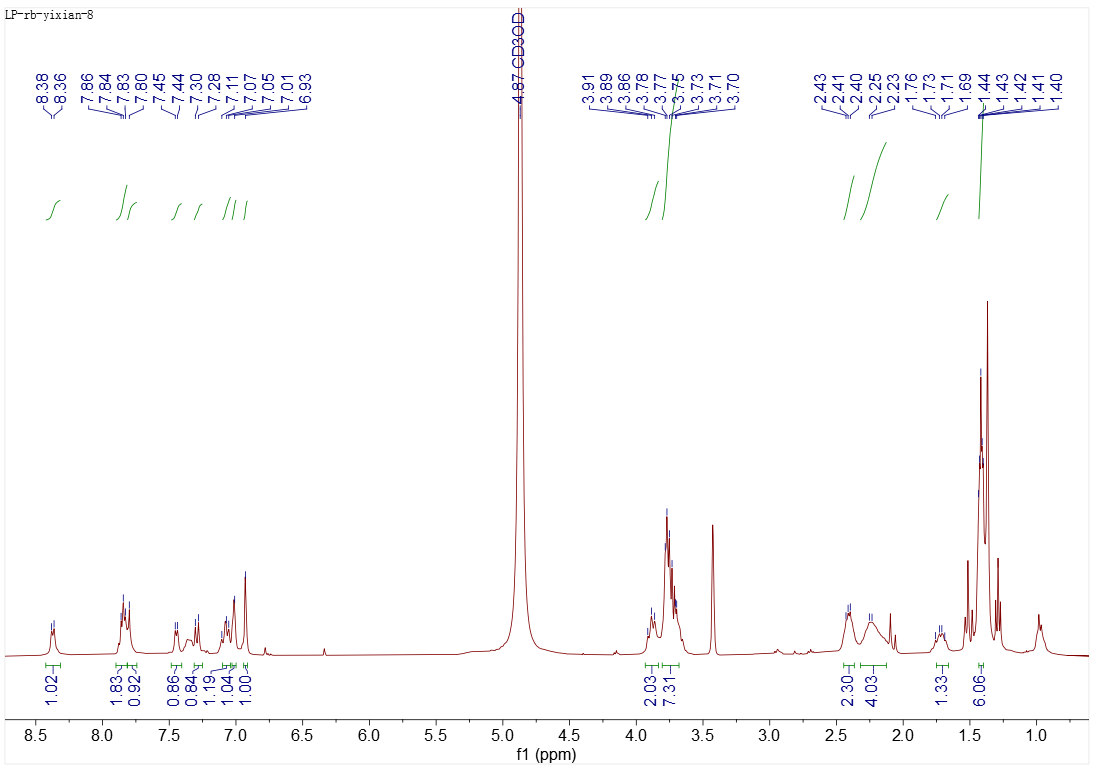


**Figure S10.** ^1^HNMR spectrum of RDQF-RB-Ac in CDCl_3_ containing 20% CD_3_OD.


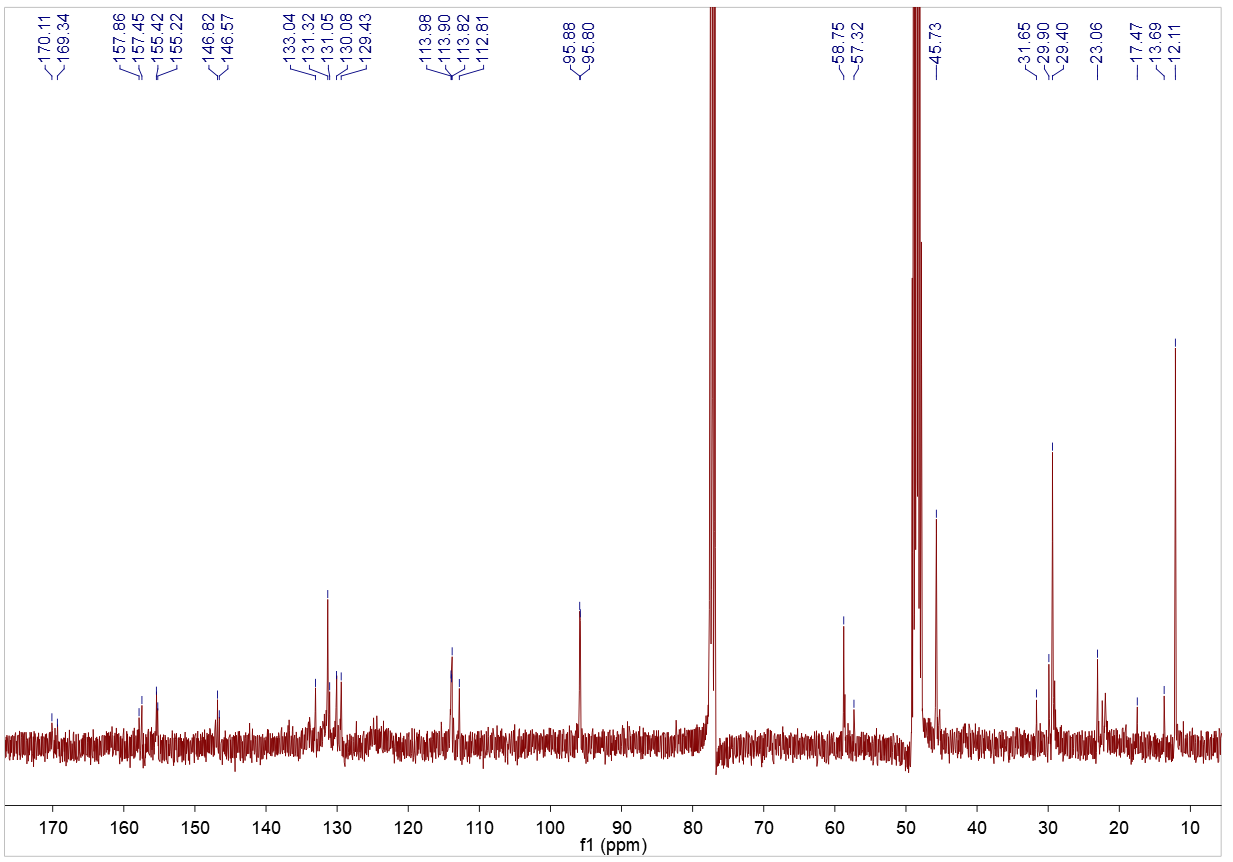


**Figure S11.** ^13^C NMR spectrum of RDQF-RB-Ac in CDCl_3_ containing 20% CD_3_OD.


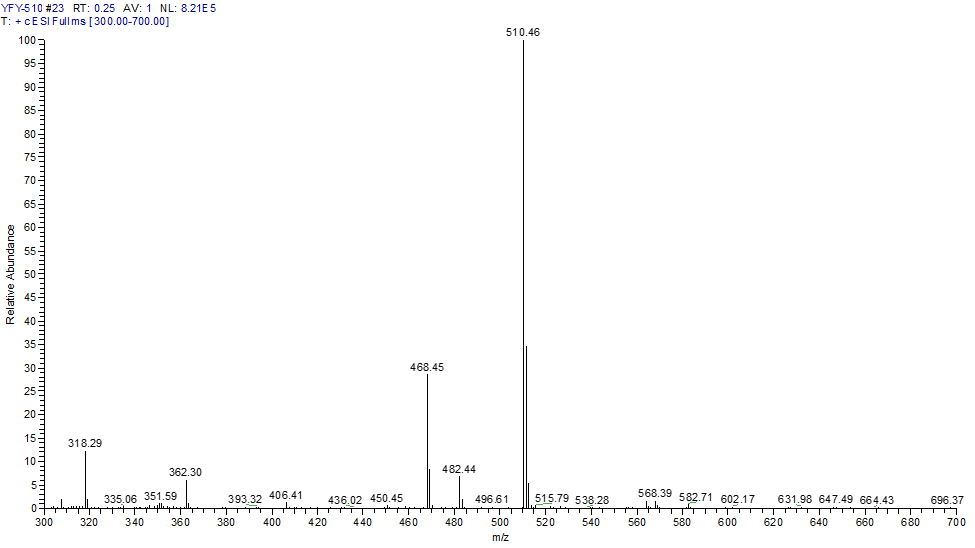


**Figure S12.** Mass spectrum (ESI) of RDQF-RB-Ac.


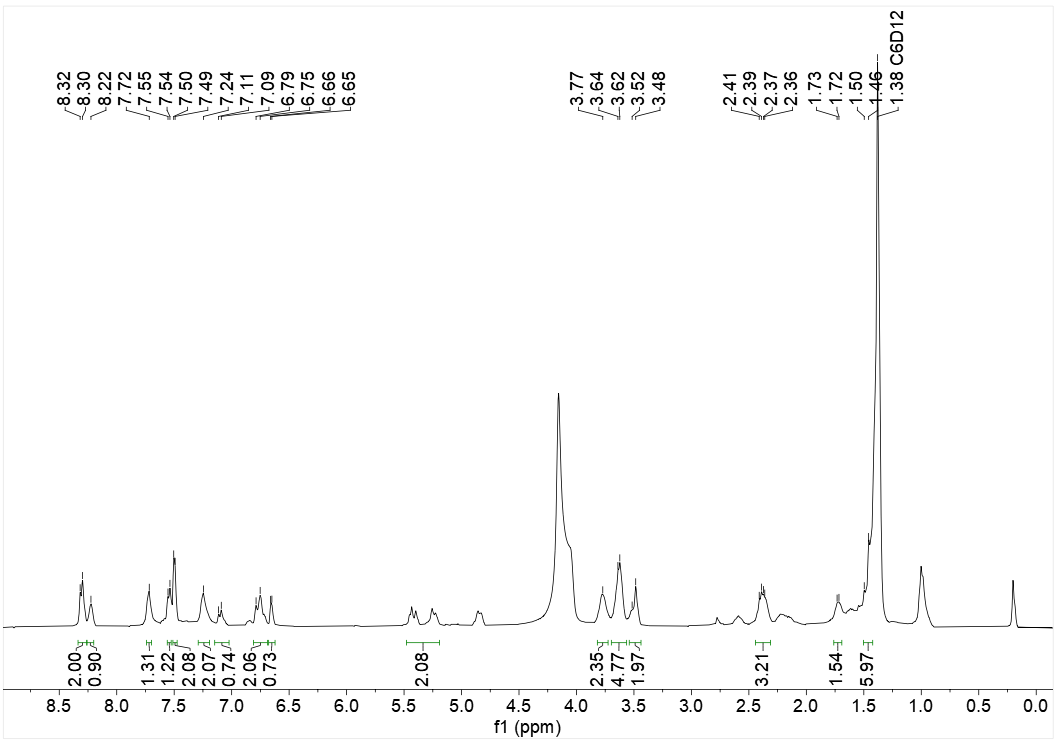


**Figure S13**. ^1^HNMR spectrum of RDQF-RB-NTR in CDCl_3_ containing 20% CD_3_OD.


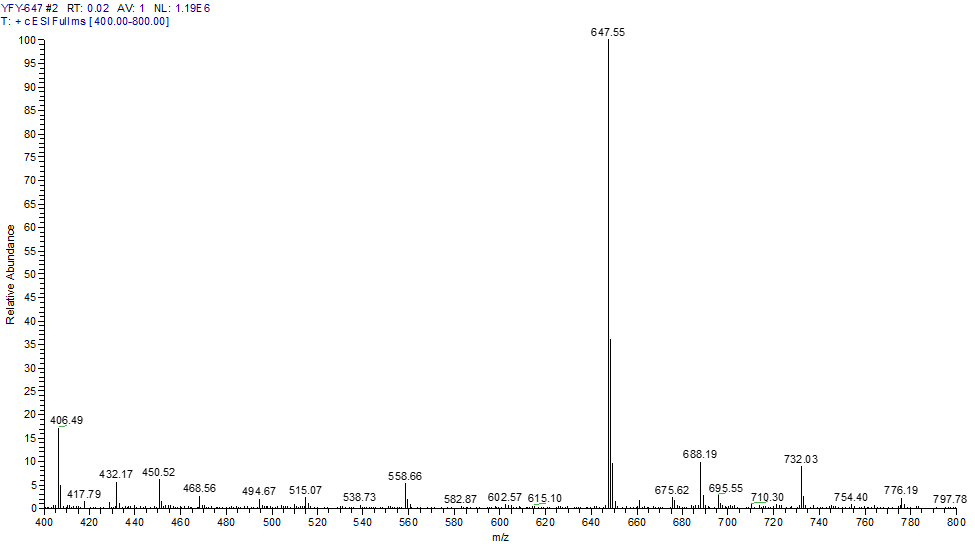


**Figure S14**. Mass spectrum (ESI) of RDQF-RB-NTR

**11. References**

1. E. Mylon, S. Roston, Am. J. Physiol. **1953**, 172, 612-616.

2. K. Rurack and M. Spieles, Anal. Chem. **2011**, 83, 4, 1232–1242.

3. R. F. Kubin and A. N. Fletcher, J. Lumin. **1982**, 27, 455– 462.

4. W. Chen, S. Xu, J. J. Day, D. F. Wang, M. Xian, Angew. Chem. Int. Ed. **2017**, 56, 16611-16615.
